# The first stygobiont species of Coleoptera from Portugal, with a molecular phylogeny of the *Siettitia* group of genera (Dytiscidae, Hydroporinae, Hydroporini, Siettitiina)

**DOI:** 10.3897/zookeys.813.29765

**Published:** 2019-01-07

**Authors:** Ignacio Ribera, Ana Sofia P.S. Reboleira

**Affiliations:** 1 Institute of Evolutionary Biology (CSIC-Universitat Pompeu Fabra), Barcelona, Spain Universitat Pompeu Fabra Barcelona Spain; 2 Natural History Museum of Denmark, University of Copenhagen, Universitetsparken 15, DK-2100 København Ø, Denmark University of Copenhagen København Denmark

**Keywords:** Diving beetles, groundwater, new species, stygofauna, troglomorphy

## Abstract

*Iberoporuspluto***sp. n.**, the first stygobiont beetle from Portugal (Dytiscidae, Hydroporinae), is described from a single female from the cave Soprador do Carvalho (Coimbra). The species is highly troglomorphic, depigmented, blind, and with elongated appendages not adapted for swimming. A molecular phylogeny based on a combination of three mitochondrial and two nuclear genes showed the new species to be sister to *I.cermenius* Castro & Delgado, 2001 from Córdoba (south of Spain), within the subtribe Siettitiina of the tribe Hydroporini. Both species are included in a clade with *Siettitiaavenionensis* Guignot, 1925 (south of France) and *Rhithrodytesagnus* Foster, 1992 and *R.argaensis* Fery & Bilton, 1996 (north of Portugal), in turn sister to the rest of species of genus *Rhithrodytes* Bameul, 1989, in what is here considered the *Siettitia* group of genera. We resolve the paraphyly of *Rhithrodytes* by transferring the two Portuguese species to *Iberoporus* Castro & Delgado, 2001, *I.agnus* (Foster, 1992), **comb. n.** and *I.argaensis* (Fery & Bilton, 1996), **comb. n.**

## Introduction

The knowledge of the subterranean fauna from Portugal has significantly increased over the last decade, with the description of a high number of obligate subterranean species (tripling their number) and the establishment of new biogeographic patterns (Reboleira 2012). A high number of these species are stygobiont (i.e., confined to groundwater), mostly from wells in the north of the country, where evapotranspiration is higher (Reboleira et al. 2011, 2013). They include 62 species of crustaceans, mostly asellids, syncarids and amphipods, and one species of annelid (Reboleira et al. 2013).

In this work we describe the first stygobiont species of Coleoptera from Portugal, a diving beetle of the subtribe Siettitiina (Dytiscidae, Hydroporinae, Hydroporini; type genus: *Siettitia* Abeille de Perrin, 1904). Siettitiina includes the only known European genera of Dytiscidae which have stygobiont members: *Siettitia*, with two species in France, *Iberoporus* Castro & Delgado, 2001, with one species in south Spain, *Etruscodytes* Mazza et al., 2013, with one Italian species, and *Graptodytes* Seidlitz, 1887, with the Moroccan *G.eremitus* Ribera & Faille, 2010 among several epigean members (Ribera and Faille 2010, Nilsson and Hájek 2018a). The subtribe also includes some North American stygobiont species, with an uncertain phylogenetic position (Miller et al. 2013, Kanda et al. 2016, Miller and Bergsten 2016, Nilsson and Hájek 2018b). The new species is known from a single female found in a well-studied cave in central Portugal. Despite multiple visits to the same cave no additional specimens have been found, so we describe here the species on the basis of its morphological singularity and of the molecular data that places it unambiguously among the west Mediterranean species of Siettitiina.

## Material and methods

### Taxon sampling, DNA extraction and sequencing

For the phylogenetic placement of the new species we used the datasets of Ribera and Faille (2010) and Abellán et al. (2013), with the inclusion of additional sequences (mostly nuclear genes) and taxa (Table 1). Most notably is the inclusion of *Siettitiaavenionensis* Guignot, 1925, the second oldest described stygobiont water beetle worldwide. Partial sequences of the genes COI and 18S were obtained from a larva preserved in 70% ethanol, collected in 1989 (Table 1). Other attempts to extract and sequence different larvae from the same locality collected in 1984 and 1992 (Ph. Richoux leg.) proved unsuccessful. Extractions of single specimens were non-destructive, using a standard phenol-chloroform method or the DNeasy Tissue Kit (Qiagen GmbH, Hilden, Germany). Vouchers and DNA samples are kept in the collections of the Museo Nacional de Ciencias Naturales, Madrid, Madrid (**MNCN**), the Institute of Evolutionary Biology, Barcelona (**IBE**) and the Natural History Museum of Denmark (**NHMD**).

**Table 1.** Material used in the molecular phylogeny of the *Siettitia* group of genera, with locality, collector, and EMBL accession numbers. Newly obtained sequences are in bold typeface. Nomenclature follows Nilsson and Hájek (2018a).

*N*	Species	Voucher	Locality, date, and collector	COI-5’	COI-3’	16S+	18S	H3
1	* Graptodytes aequalis *	NHM-IR206	Morocco: Debdou, Meson forestiere; 6.4.1999, I Ribera, P Aguilera, C Hernando, A Millán	**LS999725**	HM588264	AY250910	AJ850509	EF670184
2	* G. atlantis *	MNCN-AI921	Morocco: Lac Afenourir, Azrou; 29.4.2000, I Ribera	**LS999726**	HM588265	HM588602	**LS999692**	**LS999771**
3	* G. bilineatus *	MNCN-AI608	Sweden: Västerbotten prov., Åmsele, Vindelälven; 18.9.2005, AN Nilsson	**LS999727**	HM588267	HM588603	**LS999693**	**LS999772**
4	* G. castilianus *	MNCN-AI1316	Spain: Navarra, Pitillas: pond in crossroad; 21.7.2004, I Ribera, A Cieslak	HF947943	HM588268	HM588604	**LS999694**	**LS999773**
5	* G. delectus *	MNCN-AI1092	Tenerife (Spain): Chamorga, Bco. Roque Bermejo; 20.7.2006, A Castro	**LS999728**	HM588269	HM588605	**LS999695**	**LS999774**
6	* G. eremitus *	IBE-AF33	Morocco: Tiqqi, cave Doussoulile; 28.7.2008, JM Bichain et al.	**LS999729**	HM588271	HM588606	**LS999696**	**LS999775**
7	* G. flavipes *	NHM-IR40	Spain: Huelva, Almonte, poblado forestal; 26.7.1998, I Ribera	–	HM588273	AY250914	AJ318730	EF056561
8	* G. fractus *	MNCN-AI627	Spain: Córdoba, Sa. de Córdoba, Arroyo de los Arenales; 16.4.2005, A Castro	LS451100	HM588274	HM588608	LS453474	LS453168
9	* G. granularis *	MNCN-AI609	Sweden: Västerbotten prov., Åmsele, Vindelälven; 18.9.2005, AN Nilsson	**LS999730**	HM588278	HM588611	**LS999697**	**LS999776**
10	* G. ignotus *	NHM-IR531	Spain: Girona, Estanys de Capmany, 3.2001, P Aguilera	**LS999731**	HM588287	AY250915	AJ850510	EF670185
11	* G. kuchtae *	MNCN-AI177	Mallorca (Spain): Ternelles, Torrent de Ternelles; 14.10.2004, I Ribera, A Cieslak	**LS999732**	HM588288	HM588614	**LS999698**	**LS999777**
12	* G. laeticulus *	MNCN-HI16	Algeria: Algeria, Aïn Damous; 24.8.2006, S Bouzid	–	HM588300	HM588621	**LS999699**	**LS999778**
13	* G. pictus *	MNCN-AI660	Poland: Zachodniopomorsky, Dygowo: pond; 16.8.2004, I Ribera, A Cieslak	**LS999733**	HM588290	HM588615	**LS999700**	**LS999779**
14	* G. pietrii *	MNCN-DM37	Tunisia: Rd. Beja-Teboursouk, NW Teboursouk; 23.10.2001, I Ribera, A Cieslak	**LS999734**	HM588292	HM588616	**LS999701**	**LS999780**
15	* G. sedilloti sedilloti *	NHM-IR585	Cyprus; 3.2001, K Miller	LS451098	HM588294	HM588619	LS453473	LS453167
16	* G. sedilloti phrygius *	MNCN-AI111	Chios (Greece): Marmaro marsh; 19.4.2004, GN Foster	**LS999735**	HM588293	HM588618	**LS999702**	**LS999781**
17	* G. siculus *	MNCN-AH162	Sicily (Italy): Parco dei Nebrodi, Stream Trail Lago Urio; 13.6.2007, P Abellán, F Picazo	**LS999736**	HM588295	HM588620	**LS999703**	**LS999782**
18	* G. varius *	MNCN-AH160	Sicily (Italy): Parco dei Nebrodi, Stream Trail Lago Urio; 13.6.2007, P Abellán, F Picazo	**LS999737**	HM588297	HM588622	**LS999704**	**LS999783**
19	* G. veterator veterator *	MNCN-AH161	Sicily (Italy): Parco dei Nebrodi, Stream Trail Lago Urio; 13.6.2007, P Abellán, F Picazo	LS451095	HM588304	HM588625	LS453472	LS453105
20	* G. veterator behningi *	MNCN-AI774	Turkey: Düzce, Rd. to Kartalkaya from Çaydurt; 23.4.2006, I Ribera	**LS999738**	HM588303	HM588624	**LS999705**	**LS999784**
21	* Iberoporus cermenius *	NHM-IR276	Spain: Cordoba, Priego de Cordoba; 29.4.2000, A Castro	LS451107	AY250958	AY250918	AJ850511	EF670186
22	*I.pluto* sp. n.	IBE-AN151	Portugal: Soprador do Carvalho; 24.10.2014, ASPS Reboleira	**LS999739**	**LS999756**	**LS999763**	**LS999706**	**LS999785**
23	* Metaporus meridionalis *	NHM-IR34	Spain: Albacete, Robledo, Ojos de Villaverde; 7.9.1997, I Ribera	–	HM588307	AY250919	AJ318739	EF670187
24	* Porhydrus genei *	IBE-RA86	Algeria: Garaet Aïn Nechma, nr Ben-Azzouz (Skikda); 29.6.2009, S Bouzid	**LS999740**	HF931320	HF931543	**LS999707**	**LS999786**
25	* P. lineatus *	NHM-IR24	England (UK): Sommerset Levels, Chilton Trinity; 4.7.1998, I Ribera	**LS999741**	AY250973	AY250933	AJ318743	EF670188
26	* P. obliquesignatus *	IBE-RA147	Italy: Piano Grande. Piano di Castelluccio; 20.7.2009, M Toledo	**LS999742**	HF931305	**LS999764**	**LS999708**	**LS999787**
27	* P. vicinus *	MNCN-AH113	Portugal: Cercal, ephemeral pond btw. Cercal and Vilanova; 24.1.2008, I Ribera	**LS999743**	HF931132	HF931350	**LS999709**	**LS999788**
28	* Rhithrodytes agnus *	MNCN-AI1007	Portugal: Viana do Castelo, N Ponte de Lima, W Labruja; 28.5.2006, H Fery	**LS999744**	HF931143	HF931362	**LS999710**	**LS999789**
29	* R. argaensis *	MNCN-AI179	Portugal: Serra de Arga, Pools on summit; 9.5.2005, DT Bilton	HF948005	HF931183	HF931405	**LS999711**	**LS999790**
30	* R. bimaculatus *	IBE-RA727	Spain: Huesca, Aragués del Puerto; 23.7.2011, I Esteban	**LS999745**	**LS999757**	**LS999765**	**LS999712**	**LS999791**
31	* R. crux *	MNCN-AI302	Italy: Alessandria, stream; 2.5 km S Praglia; 18.10.2002, I Ribera, A Cieslak	LS451084	HF931187	HF931410	LS453475	LS453108
32	* R. numidicus *	MNCN-DM34	Tunisia: Rd. Tabarka-Aïn-Draham, stream Aïn-Draham; 23.10.2001, I Ribera, A Cieslak	–	**LS999758**	**LS999766**	**LS999713**	**LS999792**
33	* R. sexguttatus *	NHM-IR183	Corsica (France): Porto-Vecchio: l’Ospedale; 18.9.1999, I Ribera, A Cieslak	–	AY250975	AY250936	AJ850513	EF670190
34	* Siettitia avenionensis *	MNCN-AI897	France: Barbentane; 22.2.1992, J Dalmon	–	**LS999759**	–	**LS999714**	–
35	* Stictonectes abellani *	IBE-PA312	Spain: Ciudad Real, PN Cabañeros; 7.7.2008, A Millán and col.	LS451083	HF931298	HF931530	LS453469	LS453169
36	* S. azruensis *	NHM-IR661	Morocco: Moyen Atlas, nr. Azrou, Col du Zad; 16.4.2001, Pellecchia, Pizzetti	**LS999746**	AY250979	AY250940	**LS999715**	**LS999793**
37	* S. canariensis *	IBE-AF114	Gran Canaria (Spain): Barranco Güigüi grande; 1.4.2008, J Hájek, K Kaliková	**LS999747**	HF931113	HF931330	**LS999716**	**LS999794**
38	* S. epipleuricus *	MNCN-AH73	Portugal: Serra de São Mamede, Portalegre: r. Caia; 25.7.1998, I Ribera	**LS999748**	**LS999760**	**LS999767**	**LS999717**	–
39	* S. escheri *	MNCN-AH107	Morocco: Asilah, rd. N1, stream ca.; 4 km S Asilah; 27.3.2008, I Ribera, P Aguilera, C Hernando	**LS999749**	HF931130	HF931349	**LS999718**	**LS999795**
40	* S. formosus *	MNCN-AH108	Morocco: Asilah, rd. N1, stream ca.; 4 km S Asilah; 27.3.2008, I Ribera, P Aguilera, C Hernando	**LS999750**	HF931131	**LS999768**	**LS999719**	**LS999796**
41	* S. lepidus *	MNCN-AI632	Spain: Córdoba, Sierra Morena, cta. Villaviciosa; 16.4.2005, A Castro	**LS999751**	**LS999761**	**LS999769**	**LS999720**	**LS999797**
42	* S. occidentalis *	NHM-IR529	Portugal: Algarve; 2001, P Aguilera	–	AY250980	AY250942	–	**LS999798**
43	* S. optatus *	MNCN-AI1089	Spain: Jaén, Sierra de Cazorla, cta. Del Tranco; 3.8.2006, A Castro	**LS999752**	**LS999762**	**LS999770**	**LS999721**	**LS999799**
44	* S. optatus *	NHM-MsC	Corsica (France): Porto-Vecchio: l’Ospedale; 18.9.1999, I Ribera, A Cieslak	–	AY250981	AY250943	AJ850514	EF670192
45	* S. rebeccae *	MNCN-AH72	Portugal: Serra Estrela, Sabugueiro, r. above village; 12.5.2005, I Ribera	**LS999753**	FR851207	FR851208	**LS999722**	**LS999800**
46	* S. rufulus *	MNCN-AI1299	Sardinia (Italy): Road from Óschiri to Mount Limbara; 17.10.2006, GN Foster	**LS999754**	HF931179	HF931400	**LS999723**	**LS999801**
47	* S. samai *	IBE-AF142	Algeria: Oued Bagrat; 24.3.2006, S Bouzid	**LS999755**	HF931119	HF931336	**LS999724**	**LS999802**

Examples of most species of Palaearctic Siettitiina were included, including all stygobiont or interstitial species with the exception of *Graptodytesaurasius* Jeannel, 1907 (Algeria), *Siettitiabalsetensis* Abeille de Perrin, 1904 (France) and *Etruscodytesnethuns*Mazza et al., 2013 (Italy). Trees were rooted in the split between *Graptodytes*+*Metaporus* Guignot, 1945 and the rest of Siettitiina, based on previous phylogenetic results (Ribera et al. 2008, Abellán et al. 2013).

Fragments of five genes in five sequencing reactions were sequenced, three mitochondrial (1) 5’ end of cytochrome c oxidase subunit 1 (COI-5, “barcode” fragment of Hebert et al. 2003); (2) 3’ end of cytochrome c oxidase subunit 1 (COI-3); (3) 5’ end of 16S RNA plus the Leucine tRNA plus 5’ end of NADH dehydrogenase subunit I (16S); and two nuclear fragments (4) an internal fragment of the small ribosomal unit, 18S RNA (18S) and (5) an internal fragment of Histone 3 (H3). Details on primers used are provided in Table 2. Sequences were assembled and edited with Geneious v6.0.6 (Kearse et al. 2012); new sequences (111) have been submitted to the EMBL database with accession numbers LS999692-LS999802 (Table 1).

**Table 2.** Primers used in the amplifying and sequencing reactions.

Gene	Primer	Sequence	Reference
COI-3’	Jerry (5’)	CAACATTTATTTTGATTTTTTGG	Simon et al. (1994)
	Pat (3’)	TCCAATGCACTAATCTGCCATATTA	
	Chy (5’)	T(A/T)GTAGCCCA(T/C)TTTCATTA(T/C)GT	Ribera et al. (2010)
	Tom (3’)	AC(A/G)TAATGAAA(A/G)TGGGCTAC(T/A)A	
COI-5’	Uni LepF1b	TAATACGACTCACTATAGGGATTCAACCAATCATAAAGATATTGGAAC	Hebert et al. (2004)
	Uni LepR1	ATTAACCCTCACTAAAGTAAACTTCTGGATGTCCAAAAAATCA	
16S+trnL+nad1	16SaR (5’)	CGCCTGTTTAACAAAAACAT	Simon et al. (1994)
	ND1 (3’)	GGTCCCTTACGAATTTGAATATATCCT	
	16Sb	CCGGTCTGAACTCAGATCATGT	
18S	18S 5’	GACAACCTGGTTGATCCTGCCAGT(1)	Shull et al. (2001)
	18S b5.0	TAACCGCAACAACTTTAAT(1)	
H3	H3aF (5’)	ATGGCTCGTACCAAGCAGACRCG	Colgan et al. (1998)
	H3aR (3’)	ATATCCTTRGGCATRATRGTGAC	

### Phylogenetic analyses

Edited sequences were aligned using the online version of MAFFT 7 with the G-INS-I algorithm (Katoh and Toh 2008).

BEAST 1.8 (Drummond and Rambaut 2007) was used for Bayesian phylogenetic analyses, using a molecular-clock approach for estimating divergence times. We applied a partition by genes with uncorrelated lognormal relaxed clocks to estimate substitution rates and a Yule speciation process as the tree prior, using GTR+I+G and HKY+I+G evolutionary models. We calibrated the tree using rates estimated in Andújar et al. (2012) for a genus of Carabidae (*Carabus* Linnaeus, 1758), in the same suborder Adephaga (rate of 0.0113 [95% confidence interval 0.0081 – 0.0147] substitutions per site per million years (subst/s/Ma) for COI-5; 0.0145 [0.01 – 0.0198] subst/s/Ma for COI-3 and 0.0016 [0.001 – 0.0022] subst/s/Ma for 16S+tRNA). Analyses were run for 100 million generations, assessing that convergence was correct and estimating the burn-in fraction with Tracer v1.6 (Drummond and Rambaut 2007). We also used a fast Maximum Likelihood (ML) heuristic algorithm in RAxML-HPC2 (Stamatakis 2006) in the CIPRES Science Gateway (Miller et al. 2010), using the same partition scheme as in BEAST with a GTR+G evolutionary model independently estimated for each partition and assessing node support with 100 pseudoreplicates with a rapid bootstrapping algorithm (Stamatakis et al. 2008).

## Results

**Figure 1.**
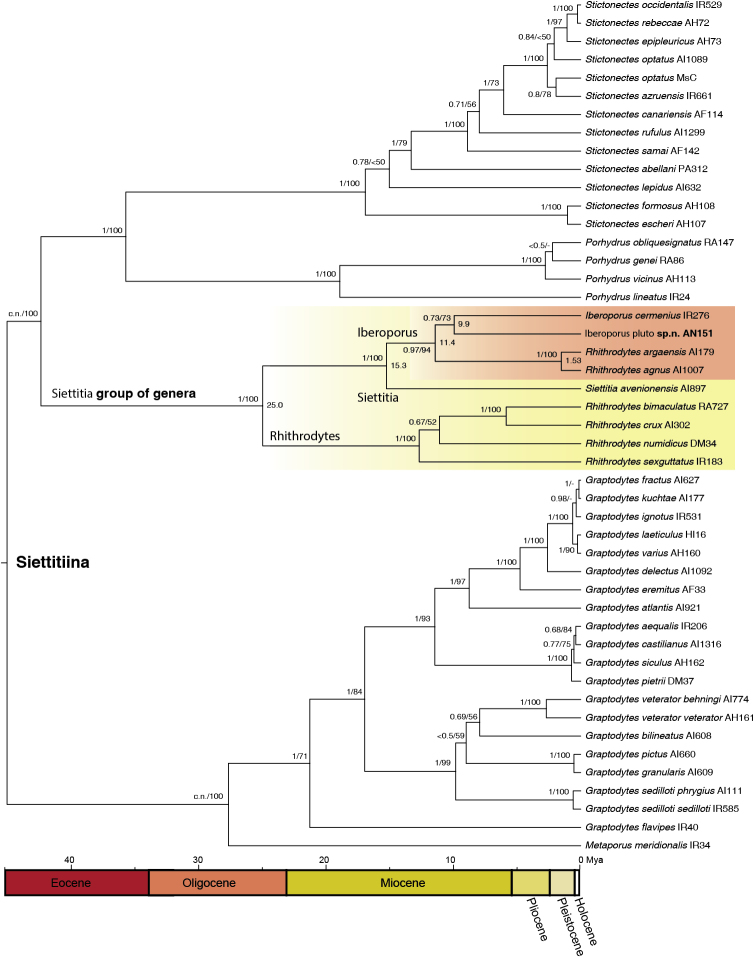
Phylogeny of the *Siettitia* group of genera, obtained with Bayesian methods. Numbers in nodes, Bayesian posterior probabilities/maximum likelihood bootstrap support (obtained in RAxML); c.n., constrained node in the Bayesian analysis. See Table 1 for details on the specimens.

The two BEAST analyses (GTR and HKY evolutionary models) resulted in identical topologies and very similar branch lengths, although convergence for GTR evolutionary models was poor for some genes (nad1, 18S), so we present here only the results of the HKY models (Fig. 1). The topology was also almost identical to that obtained with RAxML (Fig. 1).

We obtained a well-supported, well-resolved phylogeny of Siettitiina (Fig. 1). In agreement with previous results we recovered three clades, *Graptodytes*+*Metaporus*, *Stictonectes* Brinck, 1943 + *Porhydrus* Guignot, 1945, and the *Siettitia* group of genera as here defined, including *Siettitia*, *Rhithrodytes*, and *Iberoporus* (plus most likely *Etruscodytes*, see Discussion). The new species was placed as sister to *Iberoporuscermenius* Castro & Delgado, 2001 with strong bootstrap support (BS = 73%), although in the Bayesian analyses the support was lower (posterior probability, pp = 0.73). Both species were in turn sister to *Rhithrodytesargaensis* Bilton & Fery, 1996 plus *R.agnus* Foster, 1992 in a very well supported clade (BS = 94; pp = 0.97), and then to *Siettitia* (Fig. 1). All other sampled species of *Rhithrodytes* were placed as sister to this clade, rendering the genus paraphyletic. In order to preserve the monophyly of *Rhithrodytes* we thus transfer the two species to the genus *Iberoporus*, *Iberoporusagnus* (Foster, 1992) comb. n. and *Iberoporusargaensis* (Bilton & Fery, 1996), comb. n.

According to our calibration, the separation between the new species and *Iberoporuscermenius* was dated at ca. 10 Ma (95% HPD 13.4-6.9 Ma), with a similar age for the split from *I.agnus* + *I.argaensis* (11.4 Ma [15.0-8.3]), during the Tortonian (Fig. 1).

## Taxonomy

### Iberoporus pluto sp. n.

Taxon classificationAnimaliaColeopteraDytiscidae

http://zoobank.org/3F0A115A-F9F0-4AE5-95BC-E4E918FA04BB

**Figure 2.**
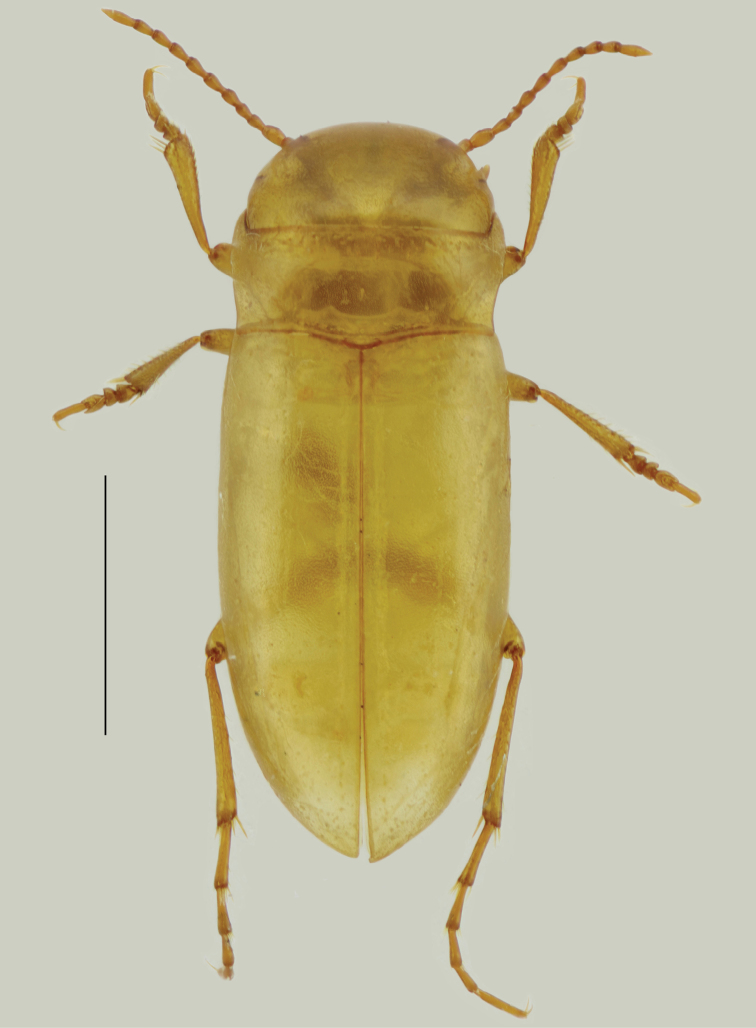
Habitus of *Iberoporuspluto* sp. n., dorsal view (holotype, after DNA extraction). Scale bar: 1 mm.

**Figure 3.**
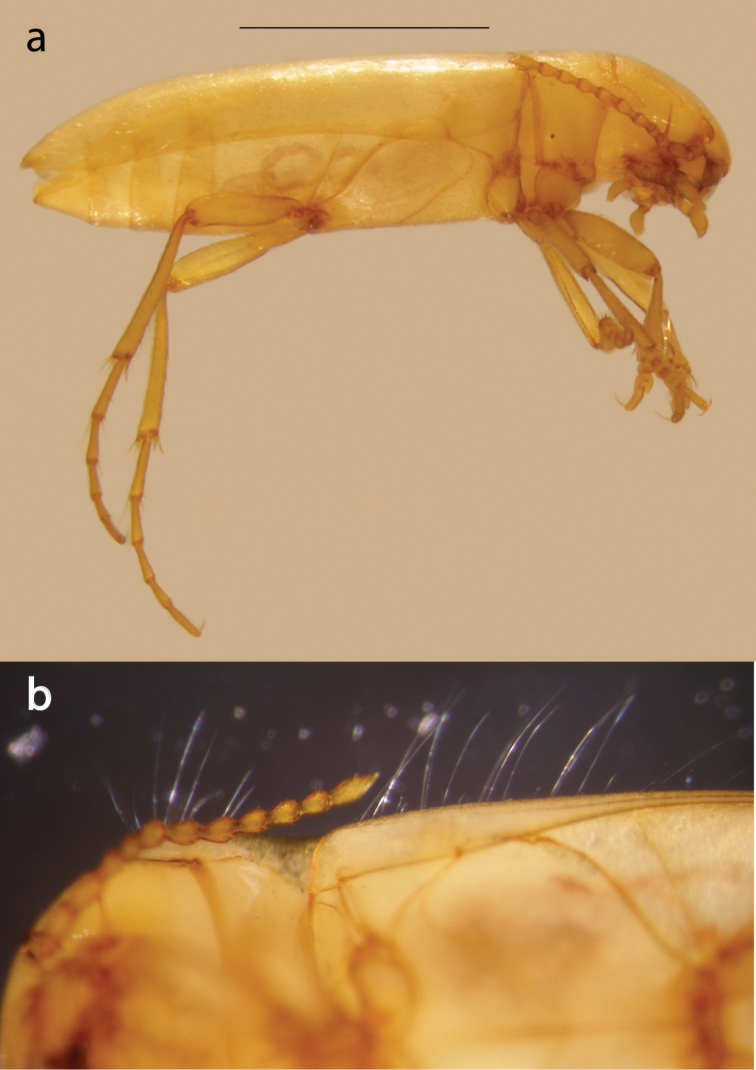
*Iberoporuspluto* sp. n., holotype. **a** Lateral view (scale bar, 1 mm) **b** Detail of the sensory setae of pronotum and elytra (both previous to DNA extraction).

**Figure 4.**
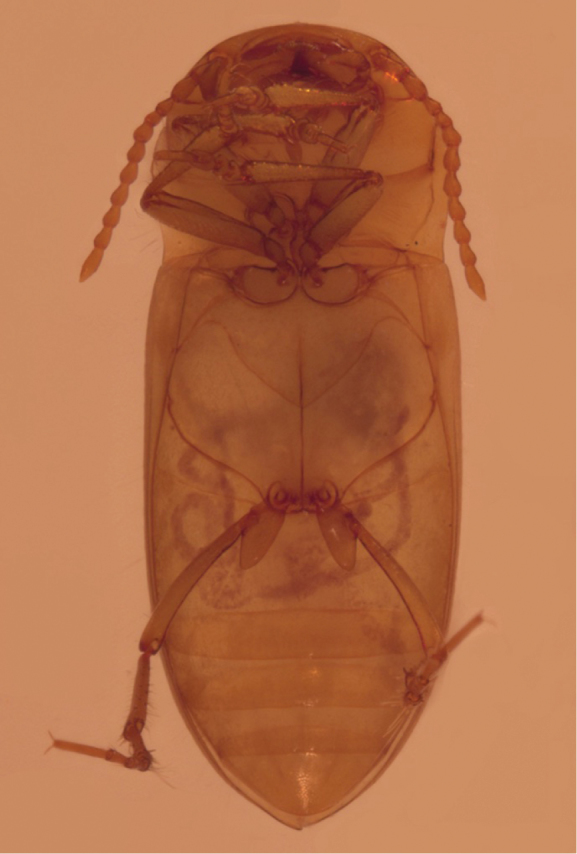
*Iberoporuspluto* sp. n., holotype, ventral view (previous to DNA extraction).

Figures 2 , 3 , 4 , 6

#### Type locality.

Portugal, Penela, Gruta Soprador do Carvalho (39°59'N, 8°23'W) (Fig. 6).

#### Type material.

**Holotype** female (NHMD) Portugal, Penela, Gruta Soprador do Carvalho, ASPS Reboleira leg., 24.X.2014, with red holotype label and DNA voucher label “IBE-AN151”.

#### Diagnosis.

A blind and depigmented species of *Iberoporus*, larger and wider than the other subterranean species of the genus, with a cordiform pronotum without lateral stria, less prominent constriction between pronotum and elytra and with a more transverse pronotum. Appendages longer and more slender, especially antennae and pro- and mesotibiae. Male unknown.

#### Description.

Body length 2.8 mm, maximum width 1.1 mm. Habitus: Body elongate, strongly parallel-sided (including pronotum and head) (Fig. 2), flattened in lateral view (Fig. 3a); in dorsal view lateral outline with a slight discontinuity between posterior angles of pronotum and base of elytra. Body and appendages uniformly pale orange (cuticle appears translucent after DNA extraction due to digestion of soft tissue).

Head (Fig. 2): Wide, anterior margin almost perfectly semicircular, deeply encased in pronotum, with two lateral dark scars in place of eyes; surface smooth, with very sparse small shallow punctures, surface weakly micro-reticulated, stronger on margins, glabrous. Antennae with ovoid pedicel, distal antennomeres conical, more elongate.

Pronotum (Figs 2, 3): Cordiform, margins sinuated, anterior part slightly wider than head, posterior part narrower than head and base of elytra; anterior margin more or less straight (except angles), angles strongly acute; posterior margin sinuated, angles acute; sides without rim, anterior margin with transverse depression with irregular row of large punctures; posterior margin with some sparse large punctures very loosely forming a row. Pronotum without sublateral stria on each side, with only a slight depression and very irregular row of larger punctures. Surface smooth, with fine shallow punctures denser on disk, with very fine microreticulation, stronger near margins, cells not contiguous; centre of disc with small longitudinal rectangular mark. Pronotum with long lateral sensorial setae (Fig. 3b).

Elytra (Figs 2, 3): almost parallel-sided on basal 2/3, apical third regularly acuminate. Sides of elytra with weak rim, not visible from above. In lateral view margin of elytra almost straight, only very weakly ascending to humeral angle in anterior quarter; epipleuron not visible until shoulders. Surface with same structure as on pronotum, with very sparse larger punctures; larger punctures forming very loose and irregular lines on elytra; more distinct near to suture and on disk. With long sensorial setae on margins (Fig. 3b). Without traces of hind wings.

Ventral surface (Fig. 4): Uniformly pale, colour similar to dorsal surface. Prosternal process lanceolate, apex acuminate; not reaching anteromedial metaventral process. Epipleuron becoming narrower short before mid-length, without oblique carina near shoulder. Metepisternum more or less triangular in shape. Metacoxal lines obsolete; joint hind margin of metacoxal processes incised; lobes of processes rounded.

Legs (Figs 2–4): long and slender, especially posterior legs. Metafemora very thin, not enlarged, regularly curved; without natatorial setae.

#### Etymology.

From “Πλούτων” (Ploutōn), the ruler of the underworld in the Greek mythology. Name in apposition.

#### Notes on the habitat.

Soprador do Carvalho is a cave with approximately 4 km of horizontal development (Fig. 7). It is the largest cave of the so-called Dueça Speleological System, located in the north-eastern part of the Sicó karst area in central Portugal (Neves et al. 2005). The subterranean stream feeds the spring of the Dueça River, a contributor to the Mondego River. The substrate of the river is mostly composed of clasts and gravel, with large clay deposits on the margins. The specimen was found in the bottom of a clay pound connected to the margin of the subterranean stream. Other invertebrate stygobionts are found in this stream, such as a new species of the asellid genus *Proasellus* and of the amphipod genus *Pseudoniphargus*, and unidentified copepods (Reboleira 2012). In the terrestrial compartment of the cave, several cave-adapted species are known: the pseudoscorpion *Occidenchthoniusduecensis* Zaragoza & Reboleira, 2018; the millipede *Scutogonaminor* Enghoff & Reboleira, 2013; the woodlice *Trichoniscoidessicoensis* Reboleira & Taiti, 2015 (which has an amphibian behaviour and can be collected inside the stream totally submerged) and *Porcelliocavernicolus* Vandel, 1946; and the dipluran Podocampacf.fragiloides Silvestri, 1932 (Enghoff and Reboleira 2013, Reboleira et al. 2015, Zaragoza and Reboleira 2018). Over recent years, the cave is being explored for tourism. This may represent a major threat, as tourists constantly trample the bottom of the subterranean stream where the new species was found.

#### Remarks.

**Figure 5.**
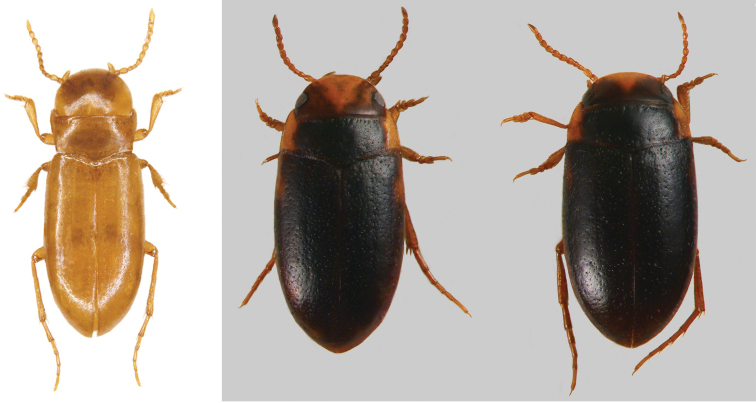
Habitus of the species of *Iberoporus*. **a***I.cermenius* (modified from Millán et al. 2014) **b***I.agnus* comb. n. **c***I.argaensis* comb. n. (both modified from Fery 2016).

*Iberoporuspluto* sp. n. is most similar in its external morphology to *I.cermenius*. Both share a similar shape of the head, a cordiform pronotum without lateral stria, and similar general appearance (Figs 2, 5a). In the absence of males of *I.pluto* sp. n. (and in addition to the genetic differences), both species can be easily separated by the body shape, larger and wider in *I.pluto* sp. n., and with a less prominent constriction between pronotum and elytra (clearly visible in *I.cermenius*) and with a more transverse pronotum. The appendages of *I.pluto* sp. n. are also longer and more slender, especially the antennae and the pro- and mesotibiae (Figs 2, 5a). *Iberoporuscermenius* has also well-defined parasutural rows on the elytra formed by large punctures, which are absent in *I.pluto* sp. n.

**Figure 6.**
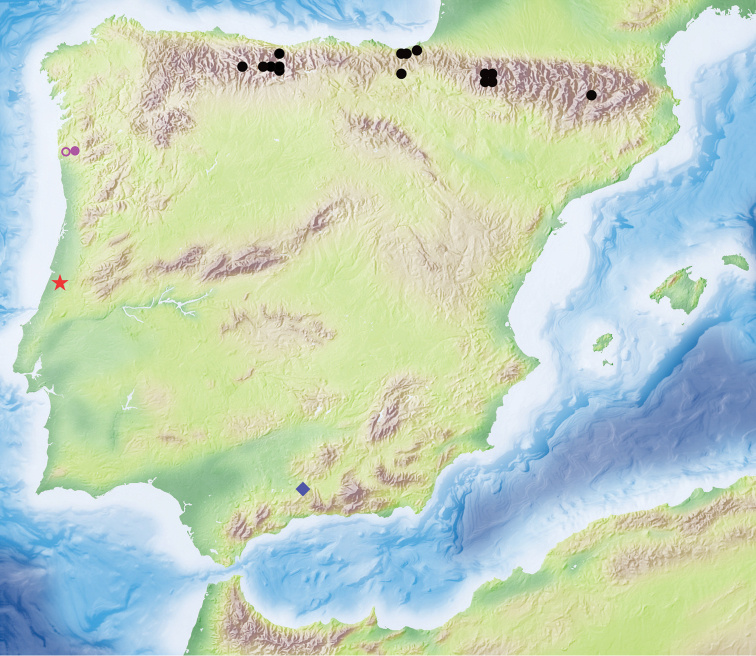
Distribution map of the Iberian species of *Rhithrodytes* and *Iberoporus*. Key: red star, *I.pluto* sp. n.; blue diamond, *I.cermenius*; filled purple circle, *I.argaensis* comb. n.; empty purple circle, *I.agnus* comb. n.; black circles, *R.bimaculatus* (data from Millán et al. 2014).

**Figure 7.**
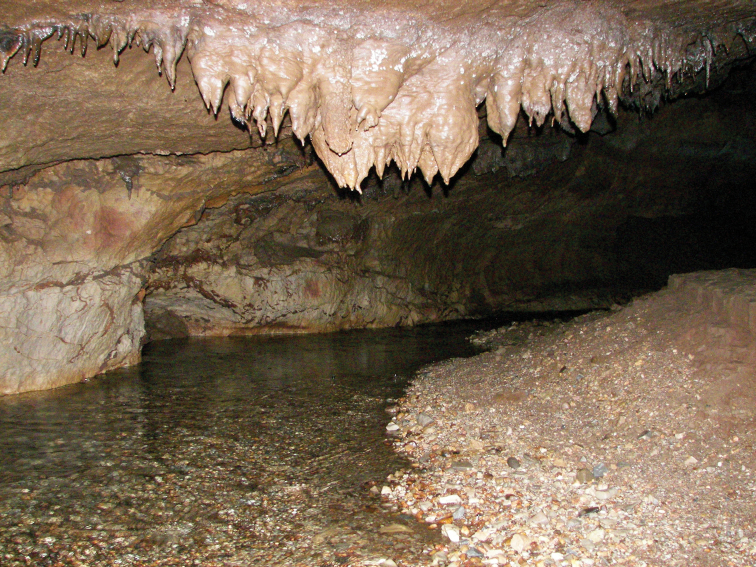
Soprador do Carvalho Cave, type locality of *Iberoporuspluto* sp. n.

## Discussion

We obtained for the first time a phylogeny of Siettitiina including a species of its type genus, *Siettitia*. Despite the incomplete data, there is strong support for the existence of a clade including *Siettitia*, *Iberoporus*, and *Rhithrodytes*, what we call the *Siettitia* group of genera. Our results also clearly demonstrate the parayphyly of *Rhithrodytes*, and the need to transfer two of the species to maintain its monophyly. The relationships between *Rhithrodytes* and the other three European stygobiont genera of Siettitiina (*Siettitia*, *Iberoporus*, and *Etruscodytes*), although widely recognised, had not been clearly established. Originally, the genus *Rhithrodytes* was erected for a group of species of *Graptodytes* (the group IV of Zimmermann 1919, or the group “crux” of Guignot 1947) with a curved apex of the median lobe of the aedeagus, a lateral stria running the whole length of the pronotum (Bameul 1989) and (as recognised later), a transverse carina in the epipleura (Fery 2013). With the exception of the epipleural carina, the rest of the characters are shared with the subterranean genus *Siettitia*, which has been for long recognised to be closely related to some of the species included in *Rhithrodytes* (e.g., *R.bimaculatus* (Dufour, 1852); Régimbart 1905, Zimmermann 1932) (Table 3).

**Table 3.** Summary comparison of some character states among the taxa of the *Siettitia* group of genera (character states of *Etruscodytes* obtained from Mazza et al. 2013).

**Character and character state**	*** Siettitia ***	*** Etruscodytes ***	***Iberoporuscermenius*, *I.pluto* sp. n.**	***Iberoporusagnus*, *I.argaensis***	***Rhithrodytes* sensu novo**
sublateral pronotal stria	long	long	absent	long	long
subhumeral epipleural carina	absent	absent?	absent	present	present
pigmentation of elytra	weak	weak	weak	strong	generally strong
eyes	absent	absent	absent	present	present
body shape, general	parallel	parallel	parallel	oval-parallel	generally oval
constriction at bases of pronotum and elytra	absent	absent	present	absent	absent
contact between prosternal process and anteromedial metaventral process	absent	present	absent	present	present
ventrites II and III	fused	not fused	fused in *I.cermenius*	not fused	not fused
elytra	fused	partly fused?	not fused	not fused	not fused

Subsequent to the description of *Rhithrodytes* two genera were described each for a single European stygobiont species: *Iberoporus* and *Etruscodytes*. *Iberoporuscermenius* shares the structure of the male genitalia with *Rhithrodytes* and *Siettitia*, but it is in particular very similar to that of *I.agnus* and *I.argaensis*. These two species (formerly in *Rhithrodytes*) have a more straight median lobe and a different shape of the apex of the parameres (Bilton and Fery 1996, Fery 2016).

The body shape of *I.agnus* and *I.argaensis* has also some similarities to the species of *Iberoporus*, parallel-sided and elongated (Figs 5b, c; see figs 12–19 in Fery 2016). *Iberoporuscermenius* shares with *Siettitia* the structure of the metacoxal processes, something that could be related to the subterranean habitat and a poor swimming ability (Castro and Delgado 2001).

*Etruscodytes*, described from a male and a female, also shares with *Rhithrodytes* and *Siettitia* the general structure of the aedeagus (note that the tip of the aedeagus in the figure of Mazza et al. 2013 seems to be broken) and the long lateral striae of the pronotum (Table 3), but nevertheless was described in a separate genus due to some morphological peculiarities (Mazza et al. 2013). Thus, according to the description by Mazza et al. (2013), the species would have (1) head wide and “subsquare” (regularly rounded in *Siettitia* and *Rhithrodytes*; although more similar to that of *Iberoporus*); (2) presence of short and flattened setae on pronotum and elytra; (3) prosternal process contacting anteromedial process of metaventrite (also in *Rhithrodytes*, not in *Siettitia* and *Iberoporus*, Table 3); (4) anteromedial process of metaventrite rounded (pointed in *Siettitia* according to Mazza et al. 2013); (5) ventrites II and III not fused (fused in *Siettitia* and *I.cermenius*, not in *I.pluto* sp. n. or *Rhithrodytes*); (6) elytra not completely fused (fused in *Siettitia*, not in *Iberoporus* and *Rhithrodytes*). Some of these characters seem to be clear autapomorphies related to the subterranean life (fusion of elytra or ventrites, particularly shaped setae, lack of lateral striae on the pronotum, lack of carina on the epipleuron), and others are of uncertain interpretation. Thus, the structure of the prosternal process is sometimes difficult to appreciate, but there do not seem to be fundamental differences between the species (note that in fig. 7 in Mazza et al. 2013 the prosternal process seems to fit below the anteromedial process of the metaventrite, which is likely an artefact), being the differences consequence of the different position of the mesocoxa (contiguous or not) and ultimately the width of the body, which in turn may depend on the habitat and ecology of the species. More data, especially molecular sequences of *Etruscodytes* and *Siettitia*, and the likely discovery of other subterranean taxa would contribute to the understanding of the evolution of this western Mediterranean lineage.

## Supplementary Material

XML Treatment for Iberoporus pluto
